# Mimicking Epithelial Tissues in Three-Dimensional Cell Culture Models

**DOI:** 10.3389/fbioe.2018.00197

**Published:** 2018-12-18

**Authors:** Núria Torras, María García-Díaz, Vanesa Fernández-Majada, Elena Martínez

**Affiliations:** ^1^Biomimetic Systems for Cell Engineering, Institute for Bioengineering of Catalonia, Barcelona Institute of Science and Technology, Barcelona, Spain; ^2^Centro de Investigación Biomédica en Red, Madrid, Spain; ^3^Department of Electronics and Biomedical Engineering, University of Barcelona, Barcelona, Spain

**Keywords:** epithelial barriers, 3D cell culture models, organoids, organ-on-a-chip, microengineered tissues, biofabrication, drug screening, disease modeling

## Abstract

Epithelial tissues are composed of layers of tightly connected cells shaped into complex three-dimensional (3D) structures such as cysts, tubules, or invaginations. These complex 3D structures are important for organ-specific functions and often create biochemical gradients that guide cell positioning and compartmentalization within the organ. One of the main functions of epithelia is to act as physical barriers that protect the underlying tissues from external insults. *In vitro*, epithelial barriers are usually mimicked by oversimplified models based on cell lines grown as monolayers on flat surfaces. While useful to answer certain questions, these models cannot fully capture the *in vivo* organ physiology and often yield poor predictions. In order to progress further in basic and translational research, disease modeling, drug discovery, and regenerative medicine, it is essential to advance the development of new *in vitro* predictive models of epithelial tissues that are capable of representing the *in vivo*-like structures and organ functionality more accurately. Here, we review current strategies for obtaining biomimetic systems in the form of advanced *in vitro* models that allow for more reliable and safer preclinical tests. The current state of the art and potential applications of self-organized cell-based systems, organ-on-a-chip devices that incorporate sensors and monitoring capabilities, as well as microfabrication techniques including bioprinting and photolithography, are discussed. These techniques could be combined to help provide highly predictive drug tests for patient-specific conditions in the near future.

## Introduction

**Figure 1:**
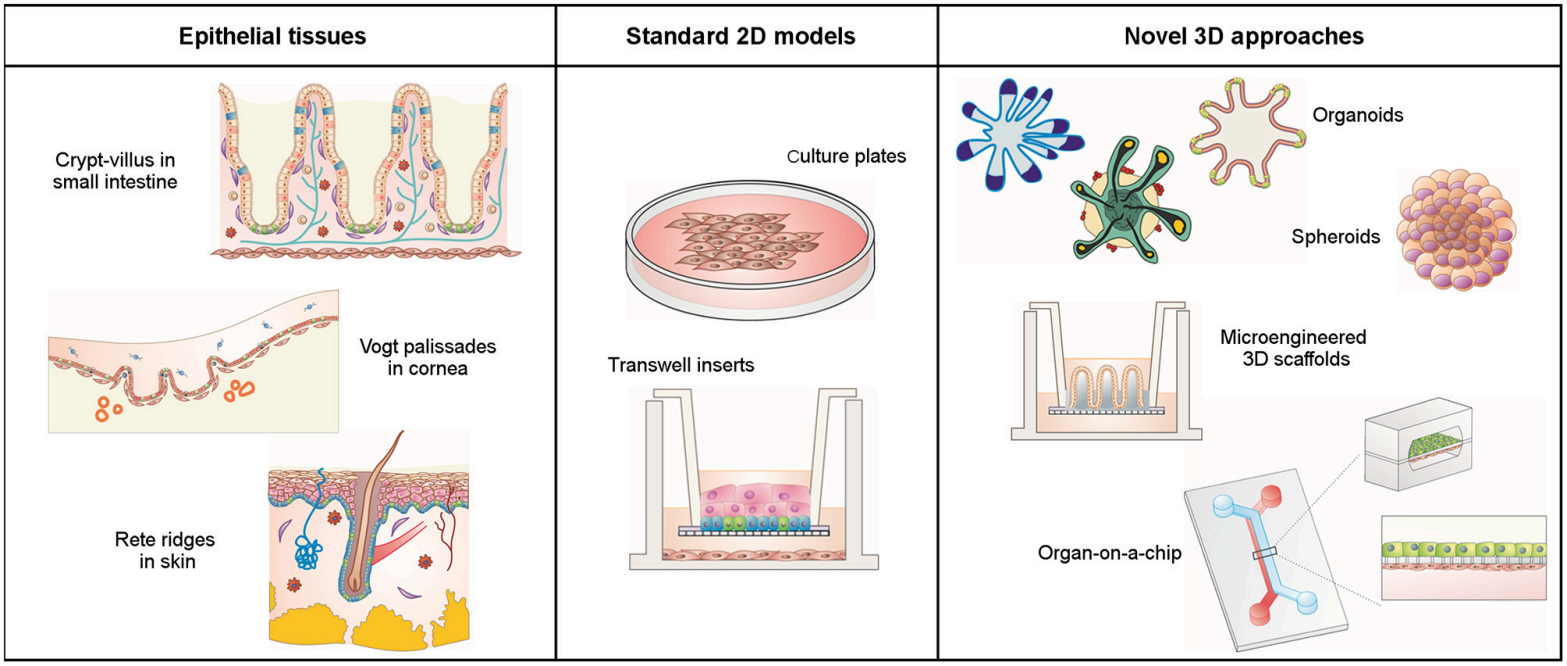
Schematic representation of epithelial tissues with their native 3D architecture, conventional 2D cell cultures, and novel 3D cell culture approaches.

Epithelial tissues are composed of cells laid out in sheets with strong intercellular bonds that form physical barriers that line the cavities of major organs (lung, skin, intestine, etc.) and protect them from external physical, chemical, and microbial insults. Epithelial cells are polarized, i.e., their apical side, facing the lumen of the organ, differs in shape and composition from the basolateral side. Epithelial cells rest on a basement membrane that acts as a growth support and as a selectively permeable layer. Besides protection, the main functions of epithelial cells include secretion, selective absorption, transcellular transport, and detection of sensation. Epithelia are actively and rapidly renewing tissues due to the presence of fast dividing adult stem cells (Crosnier et al., 2006; Vrana et al., 2013). In addition, they are a major site for carcinogenesis (Beyer et al., 2013). Many epithelial tissues have three-dimensional (3D) spatial features such as tissue widens, compact folds, invaginations, evaginations, and wavy morphologies. Such complex structures might generate biochemical factor gradients that drive the compartmentalization of the different cell types and are key determinants for organ-specific functions. Some examples are acini in the mammary gland, alveoli in the lung, Vogt palisades in the cornea, rete ridges in the skin, and crypt-villus structures in the small intestine (Figure 1). The latter are triggered by mechanical forces during morphogenesis and generate gradients of biochemical signals that spatially segregate the various tissue-forming cell types (Bollenbach and Heisenberg, 2015; Shyer et al., 2015).

Functional *in vitro* models of epithelial layers are key elements to basic research, disease modeling, drug discovery, and tissue replacement (Stange and Clevers, 2013). There is an increasing demand for *in vitro* models that are capable of capturing the complex epithelial architecture of tissues *in vivo*. Conventional preclinical models are typically two-dimensional (2D) or rely on animal models. While 2D models can provide useful information on early biological responses and are suitable for high-throughput drug screening, they have poor predictive capabilities. And while existing animal models can capture the complex physiology and interactions of *in vivo* tissues, they often fail to predict human responses due to species-specific differences. In addition, their use is often restricted due to ethical concerns. In an effort to overcome these limitations and as a kind of paradigm shift, 3D human models have emerged that are capable of capturing complex physiological responses *in vitro* (Griffith and Swartz, 2006). These new models rely on advances in cell biology, micro-engineering, biomaterials, and biofabrication.

In this study, we review the main technological strategies currently used to create 3D complex models of epithelial tissues: self-organized cell cultures, lab-on-chip devices, engineered microtissues, and various combinations of these. As the focus is typically placed on mimicking the epithelial tissue barrier properties *in vitro*, the studies reviewed here all focused on the epithelial compartment and the underlying matrix. We found that depending on the application, these engineered approaches can be extended to provide the 3D models with immunocompetent properties, microbiome, or vascularity.

## Self-Organized 3D Cell Cultures

In contrast to 2D cell culture models where cells usually grow as monolayers on flat substrates, cells in 3D culture systems self-organize as 3D aggregates, either employing a matrix as a physical support or in a scaffold-free manner. Commonly used matrices include both biologically- and synthetically-derived hydrogels such as Matrigel®, polyethylene glycol, or poly(vinyl alcohol) (Tibbitt and Anseth, 2009; Rimann and Graf-Hausner, 2012; Fang and Eglen, 2017). Based on the type of cells and their cellular organization, there are currently two types of 3D self-organized cell culture models: spheroid and organoid models. Spheroids do not need a supportive matrix to grow and are more irregularly arranged cell aggregates with a rather poor organization of relevant tissue. Organoids, on the other hand, originate from stem cells, which give rise to different organ-specific cell types and ensure the culture's high self-renewal capabilities. Organoids require a matrix to grow and possess a more ordered assembly that typically recapitulates the 3D complex tissue structures. Both 3D models have gained recent popularity as new *in vitro* tools for drug testing, disease modeling, and tissue engineering (Fang and Eglen, 2017).

Essentially, spheroids are clumps of poorly organized cells that have become a popular model in oncology research. Due to their solid spherical morphology, both oxygen and nutrients decrease toward the center, decreasing cell viability from the outer cell layers to their hypoxic and necrotic cores (Lin and Chang, 2008) (Figure 1). This feature very closely recapitulates the biochemical and cellular conditions found in most solid tumors. Tumor-derived spheroids, or tumor-spheres, have been generated from primary cancer cells (including cancer stem cells) derived from various sources such as glioma, breast, colon, ovary, and prostate tumors (Ishiguro et al., 2017). In addition to their 3D nature, the methods for generating spheroids are simple, cost-effective, highly reproducible, and adaptable, which has favored their use as *in vitro* models in the drug discovery industry in a semi-high throughput format (Youn et al., 2006; Tung et al., 2011; Vinci et al., 2012). Despite these benefits, spheroids are only poor *in vitro* models of healthy epithelial tissues, mainly due to their lack of self-renewal and differentiation properties as well as their inability to organize in tissue-like structures.

Organoids are highly organized 3D cell cultures that originated from organ specific or pluripotent stem cells with self-renewal and differentiation capabilities. When embedded in a suitable matrix and cultured with specific biochemical factors that mimic the *in vivo* stem cell niche, stem cells possess an intrinsic ability to differentiate and self-organize into 3D structures that resemble the *in vivo* organ. The culture conditions needed to generate organoids derived from intestine, skin, lung, liver, and pancreas, among others organs, employing a wealth of different cells sources from different species, are known (Rossi et al., 2018). Due to their *in vivo* resemblance in cell composition, structure and function, organoids have become the gold standard *in vitro* culture method in basic and translational epithelia research, when modeling patient-specific diseases, or as a source of autologous tissue transplantation (Yui et al., 2012; Dekkers et al., 2013; Middendorp et al., 2014).

While organoid technology undoubtedly represents a scientific breakthrough in epithelial tissue research, organoids still do not fully recapitulate all characteristics of *in vivo* epithelia. A major drawback is their 3D closed geometry, which complicates access to specific organoid compartments. For instance, the inaccessibility of the organoid-analog lumen in intestinal organoids hampers the use of conventional assays and instrumentation designed for high throughput screening studies on nutrient transport, drug absorption and delivery, or microbe-epithelium interactions (Wilson et al., 2015). In addition, the use of conventional microscopy for experimental data collection is complicated by the fact that organoids are cultured while embedded in a 3D hydrogel matrix. New strategies have been proposed to overcome these difficulties, with organoids “opened up” into flat epithelial monolayers that provide unhindered access to the luminal and basolateral compartments (Moon et al., 2014). In addition, this culture configuration has the potential to control the spatio-temporal delivery of biochemical factors through porous materials mimicking the basement membrane. When combined with 3D structures mimicking the epithelial architecture, this strategy could guide epithelial cell organization in an *in vivo-*like manner, leading to advanced organotypic 3D models (Wang et al., 2017). This will be aided by the continuing progress being made in microfluidics, biomaterials, and microfabrication techniques toward advanced 3D models.

## Lab-on-a-Chip Devices Mimicking Epithelial Tissues

Conventional approaches for differentiated epithelial cell culture are based on Transwell® systems (Rodriguez-Boulan et al., 2005) where cells form polarized monolayers on porous membranes creating independent apical and basolateral compartments, thus mimicking some basic properties of *in vivo* epithelial tissues (Figure 1). However, the highly dynamic *in vivo* environments are not represented by these static approaches (Mammoto et al., 2013). The organ-on-a-chip technology facilitates physiologically more relevant conditions and provides cells with physical and chemical stimuli by perfusing media in a laminar flow (Gayer and Basson, 2009; Cimetta et al., 2010; Thuenauer and Rodriguez-boulan, 2015). To promote cell polarization, organ-on-a-chip devices (also called microphysiological systems) usually include a porous membrane to separate two microfluidic channels. Different cells can be co-cultured on the opposite sides of this membrane, which provides a tissue-tissue interface with independent access to the cell culture chambers (Figure 1). Monitoring of the dynamic cellular responses can be achieved by incorporating biosensors and electrodes into the microfluidic device (Henry et al., 2017; Skardal et al., 2017), while its optical transparency enables direct visualization by conventional microscopy.

Several lab-on-a-chip devices, capable of representing the majority of epithelial barriers in the human body, have been designed, and kept alive and functional for several weeks. These devices can range from simple micrometer-sized chambers that simulate a specific tissue function to sophisticated “human-on-a-chip” or multi-organ microfluidic frameworks that recapitulate even complex tissue-tissue interactions (Rogal et al., 2017). One of the pioneering systems capable of fully reproducing the complex physiological functionality was the lung-on-a-chip device (Huh et al., 2010). This chip contained two apposed microchannels separated by a thin and flexible porous membrane where pulmonary epithelial cells and capillary endothelial cells were co-cultured. Cells were mechanically stimulated by the cyclic strain of breathing movements. This lung-on-a-chip system has been used for modeling respiratory diseases such as chronic obstructive pulmonary disease (Benam et al., 2016) or lung cancer (Hassell et al., 2017). Using a similar design, intestinal peristaltic movements could be mimicked with a gut-on-a-chip platform (Kim et al., 2012, 2015; Kim and Ingber, 2013). In this case, intestinal cells were exposed to fluid flow and peristaltic motion that induced villi formation and cell differentiation. This gut-on-a-chip device, along with other intestinal chips such as HuMiX, has been used to recapitulate the interplay between intestinal microbes and the epithelium (Kim et al., 2015; Shah et al., 2016). Innovative perfusable vascularized skin-on-a-chip models (Wufuer et al., 2016; Mori et al., 2017) or immune-competent models (Ramadan and Ting, 2016) have been proposed, both aiming to create more physiologically relevant skin equivalents for drug screening and disease modeling (Abaci et al., 2015; Alberti et al., 2017; van den Broek et al., 2017; Sriram et al., 2018).

A unique advantage of the organ-on-a-chip technology is its inherent ability to integrate multiple organ functions into a closed microfluidic system, which facilitates a better recapitulation *in vitro* of the human metabolism and physiology. For example, the first-pass metabolism of oral drugs can be reproduced on a gut-liver chip (Choe et al., 2017; Lee et al., 2017). “Multi-organ-on-a-chip” devices have been developed that consist of several interconnected chambers, each representing different organs of the body. These sophisticated models aim to improve predictive capabilities with regard to the spatial distribution and temporal evolution of compounds, addressing issues such as targeting, safety, and toxicity in a single device (Abaci et al., 2015; Maschmeyer et al., 2015; Skardal et al., 2017).

Although organ-on-chip devices represent a huge leap toward the generation of improved *in vitro* epithelia models, challenges such as the functional scaling or the interaction with stromal components remain (Ronaldson-Bouchard and Vunjak-Novakovic, 2018). In addition, the epithelial basement membrane is usually mimicked by a porous membrane that neither possesses the physicochemical nor mechanical properties of the native tissue matrix. Current developments try to address these limitations by exploiting advances in biomaterials and microfabrication techniques. As recent examples, full-thickness skin-on-a-chip devices used dermal matrices to represent the 3D complexity of the skin (Schimek et al., 2018; Sriram et al., 2018) and gut-on-a-chip designs included a porous scaffold that mimicked the 3D villus architecture of the small intestine (Costello et al., 2017; Shim et al., 2017). On the other hand, the combination of microfluidics with organoids from human induced pluripotent stem cells (iPSCs) or patient biopsies would likely have major implications for personalized medicine, as already exemplified by intestinal chips (Kasendra et al., 2018; Workman et al., 2018).

## Engineered Epithelial Tissues and Microtissues

Organoid technology has revealed the key role played by the matrix in guiding a cell's intrinsic self-organizing ability when forming functional tissues. However, epithelial cells are cultured on flat porous membranes of hard polymers on both Transwell® and lab-on-a-chip devices. Advances in soft biomaterials and microfabrication techniques provide new alternatives to achieve a better representation of the complex basement membrane in native epithelial tissues (Abbott, 2003; Lutolf et al., 2009; Murphy and Atala, 2014).

3D bioprinting is a relatively recent and versatile manufacturing technique that builds tissues and microtissues layer by layer using bioinks from cell-laden materials (Derby, 2012; Vijayavenkataraman et al., 2018). Bioprinted tissue constructs that faithfully recapitulate the architecture of native tissues such as skin and cornea epithelia can be fabricated in a highly reproducible manner (He et al., 2018; Sorkio et al., 2018). This approach can also be used to generate complex tubular structures such as renal proximal tubules and trachea implants (Homan et al., 2016; Bae et al., 2018). Despite its advantages, 3D bioprinting is a complex procedure that still faces many challenges such as improving cell viability and density, decreasing printing times, and increasing the printed tissue dimensions (Chang et al., 2011; Murphy and Atala, 2014). Recent advances led to the development of new bioinks, e.g., cell-derived and decellularized extracellular matrices (Fitzpatrick and McDevitt, 2015; Gopinathan and Noh, 2018) or spheroids used as individual printed units to promote *in vitro* assembly (Mironov et al., 2009; Moroni et al., 2018). Bioprinting has also evolved into a 4D technique that aims at the fabrication of time-evolving tissues by employing programmable biomaterials (Qi et al., 2013).

Lithography-based microfabrication techniques, including replica molding and photolithography, have become the standard in microelectronics to manufacture structures at cellular and subcellular scales (Whitesides, 2003). Nowadays, their application has been extended to include soft materials and to mimic 3D geometries in epithelial tissues. For instance, replica molding has been used to generate 3D microstructures that mimic the villus protrusions of the small intestine on poly(lactic-co-glycol acid) and collagen (Sung et al., 2011; Yu et al., 2012; Wang et al., 2017). Drug permeability assays have demonstrated the benefits of including the 3D tissue architecture for better predictions of the permeability found *in vivo* (Yu et al., 2012). However, replica molding of hydrogels involves a sequence of molding and demolding steps that renders this process not very amenable for mass production (Nelson et al., 2006; Sung et al., 2011; Pan et al., 2013; Cerchiari et al., 2015). In contrast, light-based polymerization approaches such as mask-based photolithography and stereolithography (SLA) can produce 3D microstructures on soft polymers in a fast, robust, and moldless manner (Tsang et al., 2007; Moroni et al., 2018). When combined with cells, the use of photoinitiator molecules and UV light might compromise cell viability, which has led to the development of new photoinitiators that are sensitive to visible light. 3D microengineered tissues generated by these techniques have been introduced into microfluidic devices or Transwell® inserts to be used as *in vitro* testing platforms (Yu et al., 2012; Costello et al., 2017; García Castaño, 2017). Recent publications also emphasized the potential of interfacing light-based microfabrication techniques with organoids to create enhanced organomimetic tissues (Schneeberger et al., 2017).

## Summary and Future Perspectives

New cell culture platforms that incorporate the unique and complex 3D architectures of epithelial tissues promise *in vitro* models with unprecedented tissue functionality. This review has highlighted recent advances in biology, biomaterials, and microfabrication techniques that could prove pivotal for the creation of these organotypic models. Advances in stem cell biology have led to the generation of organoids that recapitulate the *in vivo* 3D tissue-structure and functionality, which in itself represents a giant step toward potential applications of *in vitro* assays in the medical field (Rossi et al., 2018). For example, forskolin-induced swelling in intestinal organoids is now used as an *in vitro* test for assessing drug response in cystic fibrosis patients (Dekkers et al., 2013). In addition, current developments to establish organoids from human iPSCs in combination with novel technologies for gene editing could pave the way to personalized medicine applications. iPSC-derived organoids are used to model human organ development and disease, to test therapeutic compounds, and in cell transplantation (Shi et al., 2017). Furthermore, the 3D tissue-like cell organization provided by organoids can also be exploited to improve the functionality of organ-on-a-chip devices. By increasing the complexity of the cellular models, organ-on-a-chip approaches offer controlled and relatively simple microenvironments of sufficient biological complexity to gain greater insight into the biological mechanisms that drive disease (Bhatia and Ingber, 2014). They possess a great potential to transform drug discovery (Miranda et al., 2018) by providing human cell-based models that are capable of predicting drug delivery through epithelial barriers. In fact, pharmaceutical and biotechnological companies have already begun to incorporate these systems into their preclinical assays in an effort to improve their predictive capabilities (Ahadian et al., 2018; Cirit and Stokes, 2018). However, a better standardization and more user-friendly setups with high-throughput capabilities are needed for a broader acceptance by both industry and regulatory authorities. In this context, the development of new biofabrication techniques together with advances in biological and biomaterial research should soon allow for the development of engineered tissues and microtissues. The advantages of these structures as *in vitro* models of epithelial tissues are 2-fold: (1) they would allow the integration of non-epithelial elements that are essential for tissue function such as the immune, mesenchymal, and vascular systems (Kirkpatrick and Fuchs, 2011; Vrana et al., 2013; Battiston et al., 2014), and (2) microtissues can be easily interfaced with well plate culture formats that promise high-throughput capabilities which should promote their acceptance in the pharmaceutical and medical industries. Finally, although the key technologies reviewed here seem capable of jointly generating a set of new tools that are capable of a more accurate representation of epithelia physiological functions, the targeted applications should maintain a manageable level of complexity to provide real and meaningful impact in the biomedical and biotechnological arena.

## Author Contributions

MG-D and NT wrote the manuscript and contributed equally to this work. VF-M and EM wrote and edited the manuscript and share corresponding authorship.

### Conflict of Interest Statement

The authors declare that the research was conducted in the absence of any commercial or financial relationships that could be construed as a potential conflict of interest.
